# Transvenous Lead Extraction: The Femoral Pull‐Through Technique

**DOI:** 10.1111/jce.70138

**Published:** 2025-11-02

**Authors:** Zaki Akhtar, Manav Sohal, Raymond H. Schaerf, Mark M. Gallagher

**Affiliations:** ^1^ Department of Cardiology St. George's University Hospital NHS Foundation Trust London UK; ^2^ Smidt Heart Institute, Cedars‐Sinai Medical Institute Los Angeles California USA

**Keywords:** femoral extraction, femoral pull‐through, femoral snare, pacemaker extraction, tandem, transvenous lead extraction

## Abstract

**Background:**

Transvenous lead extraction (TLE) plays a significant role in maintaining device therapy. Conventional extraction involves advancing an extraction sheath via the implanting vein over the targeted lead, however advanced techniques involve the use of the jugular and femoral accesses which provide geometrical advantages. Femoral extraction can be used as a “bail‐out” and a primary extraction strategy but with significant challenges.

**Method:**

We present a novel technique, transferring a 20‐year‐old passive fixation atrial lead with a deployed locking stylet from the subclavian vein to the femoral site, to complete the extraction of an infected system.

**Result:**

Transfer of the lead from the implanting vein to the femoral vein, permitted linear alignment of the femoral sheath to the passive fixation atrial lead, safely enhancing the application of traction and counter‐traction. The lead was extracted without sequalae.

**Conclusion:**

The femoral pull‐through technique is safe and effective, with potential application in a range of scenarios.

## 1 Introduction

Cardiac implantable electronic devices are a key component in the management of arrhythmias and heart failure, positioning transvenous lead extraction (TLE) as a crucial procedure in their maintenance. Lead extraction is a safe and effective strategy with low procedural complications and mortality [1]. Conventional extraction techniques are based on advancing dissecting sheaths via the implanting vein over the targeted lead; occasionally the femoral approach is required to complete the procedure in a “bail‐out.” In some cases, femoral extraction has also been shown to be an effective primary strategy albeit with challenges [2]. More sophisticated techniques utilize the jugular and femoral accesses, in conjunction with the implanting vein. The Bongiorni internal transjugular approach (ITA) [3] is based on the lead pull down via the femoral and pull‐up via the jugular, whilst the Tandem technique [4] relies on simultaneous use of the femoral and subclavian for extraction. These advanced techniques exploit the geometric advantages provided by the alternative accesses to enhance safety and efficacy; evolution of TLE is essential with increasing demand and challenges.

We describe a novel technique to transfer a lead with a deployed locking stylet from the subclavian to the femoral site, permitting extraction of a passive fixation lead after 20 years dwell time by improving sheath alignment with the lead.

## 2 Case

A 71‐years‐old man with hypertension was admitted with pacemaker generator erosion. A single chamber atrial pacemaker had been implanted for sinus bradycardia in 2004 using a passive lead to the right atrial (RA) appendage. This was upgraded in 2015 to a dual chamber system with a new active fixation RA lead and a passive right ventricular (RV) lead but an attempted extraction of the original atrial lead failed.

TLE was scheduled under general anaesthesia (GA) using the Tandem approach. For each lead, the IS‐1 connector was removed, a locking stylet (Merit medical, UT, USA) was deployed to the tip, and a OneTie compression coil (Merit medical) was applied. A 13‐mm Needle's Eye Snare (NES) (Merit medical) advanced from the femoral vein, snared the targeted lead in the RA and held it taut, providing counter‐traction for the 11fr Evolution RL rotational sheath (Merit Medical). Once the rotational tool reached the RA, the lead was released from the NES and the powered sheath progressed towards the lead tip to dissect it free from the myocardium. The younger leads were extracted without difficulty, but the older RA lead remained firmly attached despite sheath advancement to the RA.

**Figure 1:**
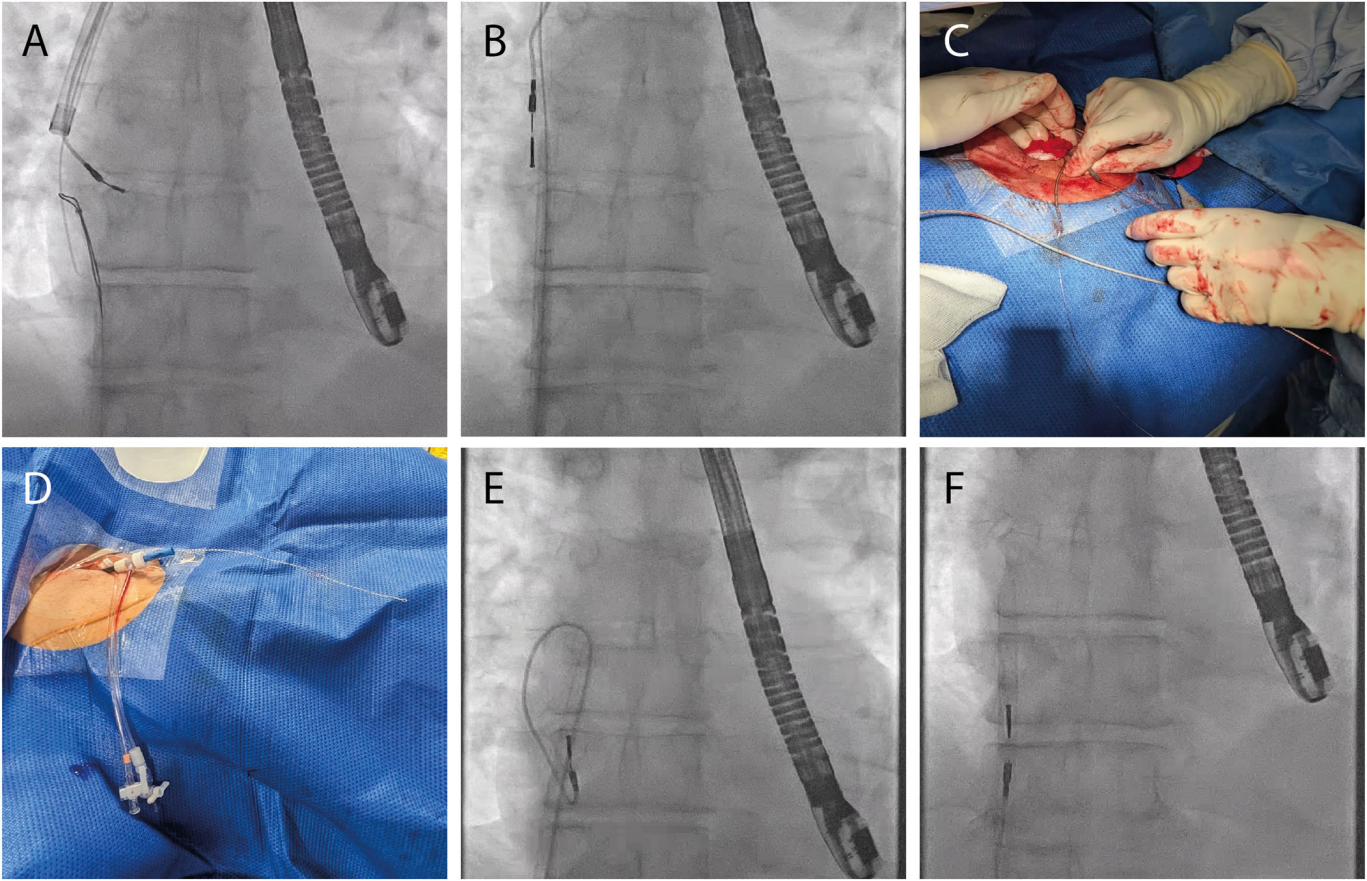
(A) The 0.035 guidewire poisoned in the right atrium (RA) from the subclavian access is snared using a loop snare via the Needle's Eye Snare (NES) outer sheath at the femoral and pulled out. (B) A long 8fr sheath is railroaded over this guidewire from the femoral to emerge at the subclavian. The dilator and wire were removed to leave the sheath to act as a “tunnel” connecting the subclavian and femoral sites. (C) At the subclavian, the locking stylet is fed in to the 8fr sheath and advanced, with the lead. (D) The locking stylet progressed via the tunnel to emerge at the femoral end where it was pulled. (E) Withdrawing the locking stylet along with the 8fr sheath straightened the lead into the NES outer sheath. (F) The NES outer sheath was perfectly aligned with the RA lead and advanced smoothly to apply countertraction to extract the lead in its entirety.

A 180 cm, 0.035 guidewire (Cordis, FL, USA) was passed from the subclavian through the Evolution outer sheath (Eos) to the RA where it was snared using a 25 mm ONESnare (Merit medical) via the NES outer sheath (NESos) located in the femoral vein. The wire was pulled into the sheath to exit at the femoral access and a long (90 cm) 8fr sheath (Roadster, Merit medical) was advanced over it via the NESos to emerge at the subclavian access site passing through the Eos. The dilator and wire were removed, leaving the Roadster sheath as a tunnel connecting the subclavian to the femoral access site. At the subclavian site, the handle of the locking stylet was fed into the Roadster and advanced to exit the femoral end of the sheath. The locking stylet, lead and sheath were withdrawn smoothly to transfer the stylet/lead unit from the subclavian to the femoral, straightening the lead entirely in the NESos. Gentle traction on the lead with counter‐traction from the NESos completed the extraction without difficulty (Figure 1).

## 3 Discussion

**Figure 2:**
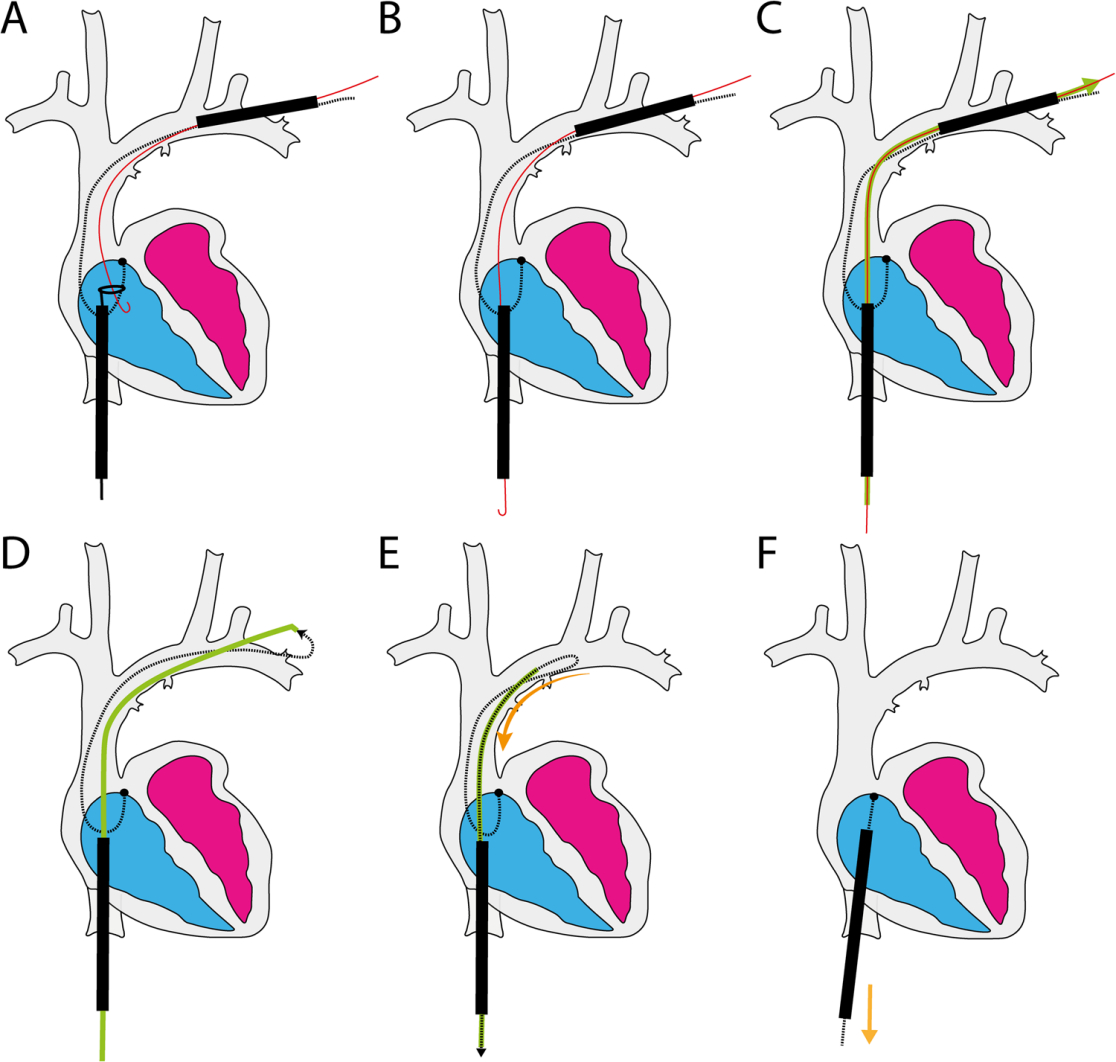
(A) Through the Needle's Eye Snare outer sheath (NESos), a loop snare was used to grasp the 0.035 guidewire (red) parked in the superior vena cava from the subclavian access, which was preserved using the outer sheath of the rotational tool. (B) The wire was pulled out through the NESos. (C) An 8fr sheath (green) was advanced over the 0.035 guidewire via the NESos, to exit at the subclavian access. (D) The 8fr sheath acted as a “tunnel” connecting the femoral and subclavian access sites. At the subclavian, the locking stylet was fed in to the “tunnel” and advanced to emerge at the femoral site. (E) The locking stylet was pulled at the femoral end to draw the stylet/lead into the “tunnel” at the subclavian; no metal remained external. The 8fr sheath and the stylet were pulled to transfer the lead from subclavian to the femoral and straightened within the NESos. (F) The NESos was advanced smoothly over the straightened lead to the tip, providing countertraction as the lead is successfully extracted with gentle traction.

We describe a novel technique to safely transfer a lead with a deployed locking stylet from the subclavian access to the femoral (Figure 2). In doing so, we straightened the lead, optimizing the alignment of sheath and lead, and permitting the advancement of the sheath to the lead tip for countertraction.

The technique is adapted from the jugular pull‐through [5], and builds upon the Bongiorni technique and the Tandem approach, which combine the superior and femoral approaches for safe and effective TLE, especially in leads of a long dwell time [4]. In our case, the Tandem approach permitted us to free the lead from the upper body veins but the rotational sheath approaching from above did not align with the lead orientation in the atrium. In comparison, extraction via the femoral access invoked a geometrical advantage: the inferior vena cava pointed the extraction sheath directly towards the RA appendage and enhanced the alignment with the atrial lead in a linear fashion. This augmented the application of traction and counter‐traction to the lead tip, reducing the risk of myocardial avulsion injury. A dissecting sheath approaching the heart through the superior vena cava (SVC) can smoothly progress to the RV apex, but it is difficult to reach a lead tip higher in the RV and impossible to meet a lead tip in the RA appendage without distorting the myocardial anatomy. Although, most cases are completed without sequalae in experienced hands, injury can be catastrophic and approximately 20% of SVC injuries occur at the SVC‐RA junction [6].

Conventional femoral extraction with the NES entails grasping of the targeted lead and engulfing it within the NES outer sheath. The lead has to ‘double‐over’ on itself as it is engulfed, which compresses the lead within the sheath and may impede progression of the countertraction exerted by the tool. Also, the proximal lead has to be withdrawn in to the vasculature to provide leeway for the sheath to progress towards the ventricular attachments and therefore the use of the locking stylet is avoided [2]. In the absence of a locking stylet, the lead is elastic with poor transmission of the traction force and less stable with the potential to de‐spiral more easily [7]. Our technique overcomes these challenges. By transferring the locking stylet to the femoral site and pulling everything straight within the NES outer sheath, we retain the strengthening effect of the locking stylet, maintain the ability to control the lead within the sheath and facilitate the advancement of the sheath to the lead tip for countertraction without difficulty (Video 1). With our method a NES outer sheath can also engulf any lead with a diameter up to 12‐fr.

### 3.1 Clinical Applications

The subclavian to femoral pull‐through is versatile and can be applied as a primary extraction method or used as a bail‐out, across all lead designs. Bracke et al found that traditional femoral extraction with the NES was challenging in RV leads of > 10 years dwell time and generally impossible with leads of > 8fr as the diameter exceeded the NES outer sheath capacity when doubled‐up [2]. Our technique overcomes these limitations by removing the need for lead doubling‐up, permitting retention of the locking stylet and preserving lead mobility within the sheath (Video 2).

### 3.2 Limitations

Patency of the inferior vena cava is essential for our method. Importantly, the technique requires freeing of the lead from the upper veins for successful transfer of the locking stylet and lead to the femoral access. For a regular patient, the overall length of the lead and locking stylet unit is sufficient to permit transfer to the femoral site, however, in tall patients a long sheath may be required to facilitate safe transfer; our patient's height was 190 cm and a 90 cm 8fr sheath was sufficient in length to act as the tunnel. Depending on the lead length, lumenless leads may not be long enough for transfer on their own and probably will require “lead extension” through the use of other tools such as a Bulldog lead extender (Merit medical).

## 4 Conclusion

The pull‐through technique allows safe transfer of a lead with a deployed locking stylet from the subclavian to the femoral access site, as well as from subclavian to jugular. It has potential in a range of scenarios.

**Video 1:** Fluoroscopy video of the femoral pull‐through technique. Using the Tandem technique, the rotational sheath progressed to the right atrium (RA) over the passive fixation atrial lead. On reaching the RA, the lead was released from the Needle's Eye Snare (NES). An 0.035 guidewire was directed to the RA from the subclavian access via the rotational tool, and snared using a loop snare from the femoral via the NES workstation; the wire exits the femoral, connectng the subclavian to the femoral. A 90 cm 8fr Roadster sheath was railroaded over the 0.035 wire from the femoral via the NES sheath, to exit at the subclavian access; the Roadster acts as a tunnel between the two access points. The free end of the locking stylet is introduced in to the Roadster sheath (at the subclavian site) and advanced until it exits the femoral end of the sheath, where it is grasped and along with the Roadster, pulled to transfer the targeted lead to the femoral. The NES sheath is then advanced over the lead in a linear fashion, to provide focal counter‐traction and extract the lead in its entirety.

**Video 2:** Femoral Pull‐Through Technique (Ex‐Vivo Demonstration). (1) A 0.035 guidewire is advanced through the Evolution outer sheath (subclavian) to the right atrium (RA). (2) Using a loop snare, the guidewire is then grasped in the RA and pulled into the Needle's Eye Snare outer sheath (NESos), until it exits the femoral access. (3) An 8‐Fr long sheath is passed over the guidewire via the NESos until it emerges from the subclavian site through the Evolution outer sheath. (4) After removing the Evolution outer sheath, the 8‐Fr long sheath remains, creating a tunnel that connects the subclavian and femoral sites. (5) The handle of the locking stylet is fed into the 8‐Fr sheath at the subclavian end. (6) Once the handle exits at the femoral end, it is grasped and pulled along with the 8‐Fr sheath, to smoothly transfer the lead from the subclavian to the femoral site. (7) With the lead now positioned in a linear alignment, the NESos is advanced over it to the attachment point. (8) Finally, focal countertraction is applied with the NESos to extract the lead.

## Data Availability

The data that support the findings of this study are available on request from the corresponding author. The data are not publicly available due to privacy or ethical restrictions.
